# A Universal Influenza Virus Vaccine Candidate Tested in a Pig Vaccination-Infection Model in the Presence of Maternal Antibodies

**DOI:** 10.3390/vaccines6030064

**Published:** 2018-09-14

**Authors:** Sun-Young Sunwoo, Michael Schotsaert, Igor Morozov, Anne Sally Davis, Yuhao Li, Jinhwa Lee, Chester McDowell, Philip Meade, Raffael Nachbagauer, Adolfo García-Sastre, Wenjun Ma, Florian Krammer, Juergen A. Richt

**Affiliations:** 1 Department of Diagnostic Medicine & Pathobiology, College of Veterinary Medicine, Kansas State University, Manhattan, KS 66506, USA; sunwoosy@gmail.com (S.-Y.S.); imorozov@vet.k-state.edu (I.M.); asally@vet.k-state.edu (A.S.D.); yuhaoli@wustl.edu (Y.L.); jinhwa@vet.k-state.edu (J.L.); cdmcdow@vet.k-state.edu (C.M.); 2 Department of Microbiology, Icahn School of Medicine at Mount Sinai, New York, NY 10029, USA; michael.schotsaert@mssm.edu (M.S.); philip.meade@icahn.mssm.edu (P.M.); raffael.nachbagauer@mssm.edu (R.N.); Adolfo.Garcia-Sastre@mssm.edu (A.G.-S.); 3 Graduate School of Biomedical Sciences, Icahn School of Medicine at Mount Sinai, New York, NY 10029, USA; 4 Global Health and Emerging Pathogens Institute, Icahn School of Medicine at Mount Sinai, New York, NY 10029, USA; 5 Department of Medicine, Icahn School of Medicine at Mount Sinai, New York, NY 10029, USA

**Keywords:** influenza, universal vaccine, pigs, vaccine-associated enhanced respiratory disease (VAERD), chimeric HA

## Abstract

The antigenically conserved hemagglutinin stalk region is a target for universal influenza virus vaccines since antibodies against it can provide broad protection against influenza viruses of different subtypes. We tested a universal influenza virus vaccination regimen based on sequential immunization with chimeric hemagglutinin (HA) containing viruses in a swine influenza virus pig model with maternal antibodies against pandemic H1N1. Vaccines were administered as live attenuated virus or inactivated influenza virus split vaccine (+/− Emulsigen adjuvant). As controls, we included groups that received trivalent inactivated influenza vaccine that contained pandemic H1N1 antigens, inactivated adjuvanted H1N2 vaccine (control group for vaccine associated enhanced respiratory disease in the pig model) or mock-vaccination. No induction of H1 head or stalk-specific antibody responses was observed upon vaccination, while responses against H3 and influenza B HA were elicited in the group vaccinated with the trivalent vaccine. Four weeks post vaccination, pigs were intratracheally challenged with pandemic H1N1 virus and euthanized 5 days after challenge. Despite the lack of detectable anti-stalk immunity, the chimeric hemagglutinin vaccine resulted in better clinical outcomes compared to control groups.

## 1. Introduction

Influenza virus infections are a major concern for veterinary and public health since zoonotic transmission of influenza viruses from birds and swine to humans are reported on a regular basis. Every year, seasonal influenza virus infections of humans occur with substantial morbidity and mortality [1,2,3]. Since influenza viruses undergo antigenic drift and shift, often resulting in novel viruses with different antigenicity, there is only limited protective immunity present in humans from previous infections or vaccinations against a newly emerging antigenic variant of the virus [4]. In addition, pandemics occur in irregular intervals and they have claimed the lives of millions of people in the past, due to of the lack of immunity in the human population against the newly emerged influenza virus strains [5]. Influenza pandemics therefore pose tremendous challenges to public health systems worldwide as experienced during the recent emergence of pandemic H1N1 (pH1N1) virus in 2009 [6,7].

The current practice for preventing influenza virus infections—vaccination with influenza A and B virus strains—is recommended annually by the World Health Organization (WHO) based on the information provided by their global influenza surveillance and response system [8]. Antibodies induced by these vaccines are neutralizing and target the globular head domain of the hemagglutinin (HA), the major surface glycoprotein of the virus. This domain is relatively plastic and has a high antigenic variability which is the reason why vaccines have to be updated every year [9,10]. In addition, even with good predictions and annual updates, mismatches between vaccine strains and circulating viruses occur periodically causing a sharp drop in vaccine effectiveness [11,12]. Therefore, vaccines that induce long lasting, broad protection against both drifting seasonal as well as pandemic influenza viruses are the focus of current research [13,14,15].

**Figure 1:**
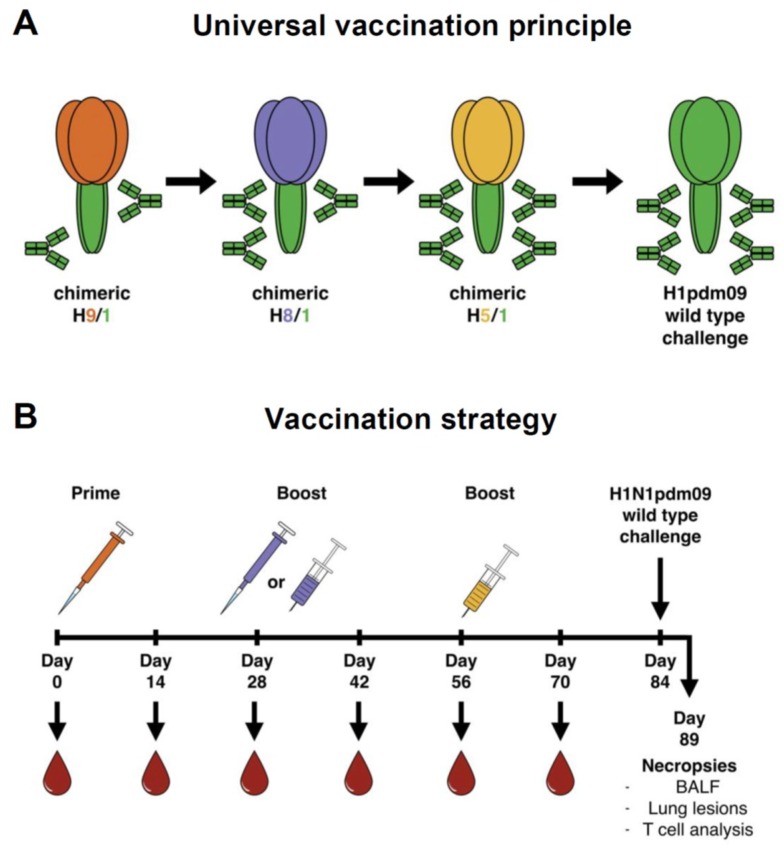
Study overview. (**A**) The universal vaccination principle is based on raising stalk HA antibodies by sequential vaccination with influenza vaccines containing HA heads of different influenza subtypes grafted on a conserved HA stalk. (**B**) Schematic representation of the experimental layout. Pipette symbol represents intranasal administration, syringe represents intramuscular injection.

In contrast to the globular head domain of the HA, the membrane-proximal stalk domain is less immunogenic and the levels of antibodies against this domain in humans are low. However, anti-stalk antibodies—both monoclonal and polyclonal—confer protection in animal models against a variety of influenza viruses with different HA subtypes due to the antigenic conservation of the HA stalk [16,17,18,19]. This makes the HA stalk domain an attractive vaccine target. One strategy for a novel, broadly protective vaccine concept is to shift the humoral immune response from the variable immunodominant HA globular head domain to the immunosubdominant HA stalk domain using sequential vaccination with chimeric HAs (cHAs) [7,14,20,21] (Figure 1A). These cHAs consist of exotic avian influenza virus HA head domains, combined with a conserved stalk domain of a seasonal virus HA [22,23]. Since humans are generally naïve to these novel head domains, but the immune system is continuously boosted by epitopes in the HA stalk when cHAs are sequentially given, this vaccination strategy can preferentially elicit antibodies against the conserved stalk domain.

Pigs are naturally susceptible to influenza A viruses, which cause considerable economic losses for the swine industry; they represent an intermediate host for the transmission of novel influenza viruses to humans and are a good model for influenza virus infection and for testing novel vaccines [24,25]. Improved, broadly protective influenza virus vaccines could also be used in pigs which would reduce the burden on the swine industry and lower the risk of zoonotic events by preventing the circulation of influenza viruses in these animals [26].

It has been reported that the use of inactivated influenza virus vaccines, HA subunit vaccines with adjuvant or M2-NP-based DNA vaccines may result in the development of vaccine-associated enhanced respiratory disease (VAERD) when vaccinated pigs are subsequently challenged with a (mismatched) influenza virus [27,28,29,30,31,32,33]. Cross-reactive anti-HA antibodies that bind exclusively to the HA2 subunit of HA (which is part of the stalk) but not to the HA1 subunit (which includes the globular head) were found in pigs that developed VAERD [34]. Since it is unclear if there is a causative role of cross-reactive HA stalk-specific antibodies in VAERD, testing of stalk-based universal influenza virus vaccination in pigs is an area of interest since it can result in the induction of HA stalk-specific antibodies. Previously, we compared protective immunity in ferrets induced by sequential immunization with chimeric-HA-based inactivated and live attenuated influenza virus vaccines [35]. In the present study, we aimed to assess the immune responses and efficacy of a universal influenza virus vaccine regimen in a swine model with natural maternal antibodies by using piglets that showed positive H1-specific antibody titers at the start of the experiment. The principle of the universal vaccine approach used in this work is depicted in Figure 1A. For a detailed description on the vaccine viruses used in this study, we refer to our previous work in ferrets, in which similar universal vaccine approaches were tested [35]. This experimental design is interesting because a widely used broadly protective vaccine might be given at a certain age and the impact of maternal immunity on vaccine efficacy, specifically for stalk-targeting vaccines, is unclear. In addition, the use of a swine model allowed us to assess whether any of the cHA-based vaccination regimens tested could induce VAERD. In this report, we show that a universal influenza virus vaccine based on the conserved stalk of HA can provide low-level protection against pH1N1 virus in pigs with maternal immunity despite the absence of a robust anti-stalk response.

## 2. Materials and Methods

### 2.1. Ethics Statement

Biosecurity level 2 (BSL-2) facilities were used for pig immunogenicity and challenge study, in compliance with the Institutional Animal Care and Use Committee at Kansas State University. All procedures for animal care and experiments were approved by the Kansas State University Institutional Biosafety Committee (IBC) and the Institutional Animal Care and Use Committee (IACUC, Protocol #: 3541).

### 2.2. Animals

A total of 30 colostrum-fed 3-week-old pigs were purchased from a commercial swine farm. All pigs had HI titers against pH1N1 virus with an average HI titer of 1:160. The pigs were randomly divided into groups with isolated pens without bias towards maternal antibody titers and acclimated for 3 days at the Large Animal Research Center at Kansas State University, Manhattan, KS, USA.

### 2.3. Experimental Design, Vaccination

The experimental groups and vaccine compositions are shown in Table 1. The pigs were separated into eight groups with three pigs for universal influenza virus vaccinated groups (G1, G1 Emul, G2, G2 Emul), six pigs for H1N2 inactivated adjuvanted influenza virus vaccine (VAERD control as previously described in [29]), six pigs for the human seasonal influenza vaccine (trivalent influenza virus vaccine: TIV), three pigs for infected control group (C-Cont) and two pigs for the non-vaccinated and non-infected group (Mock). Pigs that received universal vaccine regimens were immunized three times at 4-week intervals with appropriate vaccines administered as a 2 mL dose via the respective inoculation routes (See Figure 1B). The prime for the four universal influenza vaccine groups was done using a live chimeric influenza B virus expressing cH9/1 (H9 head from A/guinea fowl/HongKong/WF10/99 on top of an H1 stalk from A/California/04/09), via the intratracheal route (10^7^ plaque forming units (PFU) per animal). Priming with this live virus was expected to induce a baseline anti-stalk response as seen in humans, even of very young age [36]. This step also allowed us to better compare these immunization regimens with those previously tested in chimeric HA primed ferrets [35]. The first booster vaccination was then performed using 15 μg of inactivated cH8/1N1 virus (H8 globular head domain from A/mallard/Sweden/24/02 on top of H1 stalk domain from A/California/04/09, N1 from A/California/04/09 and a A/Puerto Rico/8/34 backbone) via the intramuscular route either adjuvanted (EMULSIGEN-D, MVP laboratory Inc.) or non-adjuvanted for two universal vaccine groups (groups G1 and G1 Emul). The other two groups (groups G2 and G2 Emul) were booster vaccinated with 2 × 10^7^ PFU of LAIV (A/Leningrad/134/17/57, “Leningrad” backbone; [37]) expressing the same cH8/1N1 as the IIV. The LAIV was administered in a 2 mL volume via the intranasal route. For the second boost, a dose of inactivated chimeric influenza A virus expressing cH5/1 (H5 head domain from A/Vietnam/1203/04 on top of an H1 stalk domain from A/California/04/09, N1 from A/California/04/09 and a A/Puerto Rico/8/34 backbone) equivalent to 15 μg of hemagglutinin [38] was administered in a 2 mL volume intramuscularly with (groups G1 Emul and G2 Emul) or without (groups G1 and G2) adjuvant. UV-inactivated H1N2 virus (32 HA units) with adjuvant and human seasonal vaccine (TIV) containing 15 μg of inactivated pH1N1 with adjuvant were given in a 2 mL volume intramuscularly as a prime and booster 4 weeks apart for group G3 and G4, respectively (see Table 1). Viruses for IIV generation were grown in 10-day-old embryonated eggs, purified from allantoic fluid via ultracentrifugation and inactivated with 0.03% formalin. LAIV was also grown in 10-day-old embryonated eggs and was based on the Leningrad backbone [35].

### 2.4. Challenge and Sample Collection

Four weeks after the last vaccination, pigs were challenged intratracheally with 3 × 10^6^ TCID_50_/pig of A/California/04/2009 (CA09) in 2 mL of minimum essential medium (MEM) which was the rescued pandemic H1N1 virus (see Figure 1B). Intratracheal challenge is the most reliable way for obtaining typical disease after experimental influenza challenge [39], probably due to virus receptor distribution in the swine respiratory tract [40]. Pigs were observed daily for clinical signs and were euthanized on day 5 post challenge (dpc) to evaluate viral load and lung scores. Blood samples were collected every other week during the vaccination period, prior to challenge and 5 dpc for serological analysis and isolation of PBMCs. Nasal swabs were taken on 0, 1, 3 and 5 dpc to measure the nasal shedding of virus. At necropsy, BALF was collected for virus titrations and cell-mediated immunity analysis and the right cardiac lobe of lung, trachea and nasal turbinate were collected for histopathology.

### 2.5. Virus Titration of Nasal Swabs and BALF

Virus titration of nasal swabs and BALF was performed on Madin-Darby canine kidney (MDCK) cells as previously described in detail [41]. Briefly, nasal swab samples were vortexed and centrifuged for 10 min at 704 rcf and followed by passing the supernatant through 0.45 μm filter. BALF samples that were collected by lavaging lungs with 100 mL minimum essential medium (MEM) with 1% of Penicillin, Streptomycin and Neomycin antibiotic mixture (Thermo Scientific, Waltham, MA, USA) were centrifuged and filtered as described above. All samples to measure the virus titer were tenfold serially diluted in serum-free MEM with 1 μg/mL of tosyl phenylalanyl chloromethyl ketone (TPCK) treated trypsin (Thermo Scientific, Waltham, MA, USA). Each diluent was plated onto serum-free MEM washed confluent MDCK cells in 96-well cell culture plates. MDCK growth medium composed of MEM, 5% of fetal bovine serum (FBS, Atlanta Biologicals, Flowery Branch, GA, USA), 1% of Penicillin, Streptomycin and Neomycin antibiotic mixture (Thermo Scientific, CA), 1% of MEM vitamins (Thermo Scientific, Waltham, MA, USA) and 1% of l-glutamine (Thermo Scientific, Waltham, MA, USA). Plates were evaluated for cytopathic effect (CPE) between 48 and 72 h post-infection. At 72 h post infection the medium was discarded and the plates were washed with PBS and dried. The plates were fixed with 100 μL of methanol for 5 min at room temperature and washed three times with phosphate buffered saline (PBS). The anti-influenza A nucleoprotein (NP) monoclonal antibody (ATCC#HB-65; H16-L10-4R5) was added into each well and incubated for 1 h at room temperature. Three-amino acid-9-ethylcarbazole (AEC) solution was used for staining. A TCID_50_ was calculated for each sample using the Reed and Muench method [42].

### 2.6. Pathology

At necropsy, the percentages of macroscopic lesions, defined as purple-red consolidation typical of a swine influenza virus infection on the lung surfaces, were determined for all lung lobes by an experienced veterinarian. A mean value was calculated for the seven pulmonary lobes of each pig as previously described [41,43]. Samples of distal right cardiac lung lobe, trachea (upper, medium and lower parts) and ventral nasal turbinates from each pig were placed in 10% neutral buffered formalin. Standard hematoxylin and eosin (H&E) slides and immunohistochemistry (IHC) for influenza nucleoprotein (NP) were done on 5 μm formalin-fixed, paraffin-embedded tissue sections. Historically, the cardiac lobe typically presents with pathologic lesions representative of the overall pulmonary pathology [44]. IHC for viral antigen was conducted with rabbit anti-H1N1 NP polyclonal antibody (A01506-100, GenScript, Piscataway, NJ, USA) on a Leica Bond-Max Autostainer (Leica Biosystems, Buffalo Grove, IL, USA) using Leica Biosystems reagents. Briefly, the tissue sections were placed on positively charged slides, de-paraffinized and rehydrated; antigen was retrieved with EDTA pH 9.0 buffer, blocked with 3% hydrogen peroxide diluted in distilled water and then incubated first for 15 min at room temperature with the primary antibody and then for 25 min at room temperature with anti-rabbit polyclonal-horse radish peroxidase (HRP) conjugated-IgG with intermediary rinses with Bond Wash Solution. Finally, the slides were washed in distilled water, visualized with 3,3′-diaminobenzidine (DAB) then counterstained with hematoxylin. Both, the H&E stained tissues and anti-influenza IHC were reviewed by a veterinary pathologist in a blinded fashion after optimization of the assay with control tissues.

For scoring the histopathology, a modified version of the scoring approach based on Henningson et al., 2015, was used [45]. This enabled us to score epithelial changes and severity of inflammation in the examined tissues. Lung scoring took into account percentage of parenchyma involved in the disease process as well as the degrees of peribronchial/peribronchiolar cuffing and interstitial pneumonia. Tracheal scoring included both the degree of inflammation (tracheitis) as well as epithelial lesions. A similar approach was used for the nasal cavity, rhinitis and epithelial lesions. In the computation of the final composite histopathology score for each pig, the % lung involvement score, which includes both the epithelial changes and inflammation in the examined lung field section, was weighted twice compared to all the other scores. The maximum possible score was 25. For detailed histopathology score descriptions, see Table 2. Tissues were determined to be positive or negative for influenza antigen by IHC. All microscopic images were captured with a BX46 light microscope equipped with a DP25 camera (Olympus; Tokyo, Japan) using CellSens Standard version 1.12 (Olympus) then further color calibrated using ChromaCal software version 2.5 (Datacolor Inc., Lawrenceville, NJ, USA) as per manufacturer’s instructions and published recommendations.

### 2.7. HI Assay

Sera were tested by an HI assay to assess the humoral immune response against A/Netherlands/602/2009 which is antigenically similar to A/California/04/09. Sera were diluted 1:4 in reconstituted receptor destroying enzyme (Denka Seiken, Tokyo, Japan) stock and incubated overnight at 37 °C. Three volumes of 2.5% sodium citrate solution were added to one volume of sera before a final incubation at 56 °C for 30 min to eliminate nonspecific inhibitors that might interact with influenza viruses. Post incubation, two original sera volumes of PBS were added to bring the final dilution of sera to 1:10. Stocks of A/Netherlands/602/2009 were diluted to 8 HA units/50 μL. Two-fold dilutions of 25 μL of RDE-treated sera diluted in PBS were mixed with 25 μL of viral stock in V-well microtiter plates and allowed to incubate at room temperature for 30 min. Afterwards, 50 μL of 0.5% chicken red blood cells was added to each well and the microtiter plates were incubated at 4 °C for 1 h, after which the HI titer was determined.

### 2.8. ELISA

Recombinant proteins including cH6/1 (H6 head from A/mallard/Sweden/81/02 and stalk domain from A/Puerto Rico/8/34), H1 HA from A/California/04/09, H3 HA from A/Perth/16/09, and B HA from B/Massachusetts/2/12 were produced as described before [46,47] and used as substrates. Flat-bottom Immulon 4 HBX plates 96-well plates (Thermo Scientific) were coated with 50 μL of protein diluted in ELISA coating buffer (pH 9.4) per well, at a concentration of 2 μg/mL and refrigerated at 4 °C overnight. Coating buffer was discarded and wells were blocked for 1.5–2 h at room temperature with 220 μL of blocking solution (PBS containing 0.1% Tween 20 (T-PBS), 0.5% milk, 3% goat serum (Gibco)). The blocking solution was discarded and 100 μL of serum diluted in blocking solution was added at a starting dilution of 1:400, and serially diluted 1:2 in blocking solution. After 2–3.5 h, the diluted sera were removed from the plates and the plates were washed three times with 330 μL T-PBS. The plates were then incubated for another 50–80 min at room temperature with 50 μL secondary antibody solution HRP-labeled anti-swine antibody (A5670, Sigma) diluted 1:15,000 in blocking solution. The plates were washed again four times with a shaking step and developed with 100 μL of SigmaFast o-phenylenediamine dihydrochloride (OPD; Sigma) per well. The developing process was stopped after 10 min with 3 M hydrochloric acid (HCl) and the reaction was read at an absorbance of 490 nm with a Synergy H1 hybrid multimode microplate reader (BioTek). Area under the curve analysis of absorbance data was performed in GraphPad Prism as described below.

### 2.9. Immune Cell Isolation

Five milliliters of anticoagulated blood were layered onto 3 mL of Histopaque-1077 (Sigma-Aldrich) and lymphocytes were isolated from the plasma/Histopaque-1077 interface after centrifugation (10 min, 400×rcf) at room temperature. Cells were washed by adding PBS until a volume of 10 mL was reached and resuspended in restimulation medium (Roswell Park Memorial Institute (RPMI)-1640 supplemented with 10% FBS, l-glutamine and antibiotics) after centrifugation (10 min, 250×rcf). Splenocytes were isolated by forcing 1cm^3^ of splenic tissue over a 70 μm mesh cell strainer (Corning, Oneonta, NY, USA). Red blood cells were lysed by resuspension of cells in 5 mL of NH_4_Cl solution before resuspending immune cells in 5 mL of restimulation medium.

### 2.10. IFNγ ELISpot

Quantification of IFNγ-secreting cells by ELISPOT was performed according to the immuno plate manufacturer’s protocol (R&D Systems, Minneapolis, MN). Briefly, 96-well immuno-plates pre-coated with sterile monoclonal anti-IFNγ antibodies were blocked with restimulation medium. Immune cells (10^5^) were plated in 100 μL of culture medium supplemented with UV-inactivated whole virus (same as challenge pH1N1 virus, equivalent of 10^4^ PFU/well) or medium only as negative control. After 16h of restimulation, plates were washed with wash buffer provided with the ELISpot assay kit. IFNγ bound to the plates was detected by a biotinylated polyclonal anti-IFNγ antiserum. 5-bromo-4-chloro-3-indolyl phosphate/nitro blue tetrazolium (BCIP/NBT) chromogen substrate for alkaline phosphatase conjugated to streptavidin resulted in the formation of spots at places where immune cells secreted IFNγ during virus-restimulation. The spots were counted using an ImmunoSpot S6 Micro Analyzer (Cellular Technology Ltd. (CTL), Shaker Heights, OH, USA) with ImmunoCapture 6.4 software (CTL).

### 2.11. Intracellular Cytokine Staining (ICS) and Flow Cytometry

For staining of intracellular IFNγ, 2 × 10^6^ immune cells were plated in 200 μL of restimulation media supplemented with UV-inactivated whole virus (same virus as challenge pH1N1 virus, equivalent of 10^5^ plaque forming units) and Golgiplug (1 μL/mL Brefeldin A, BD). Cells were stained for surface markers with CD3ε-PE-Cy7 (clone BB23-8E6-8C8, BD Pharmingen, San Jose, CA, USA), CD4-PERCP-Cy5.5 (clone 74-12-4, BD Pharmingen, San Jose, CA, USA), CD8a-FITC (clone 76-2-11, BD Pharmingen, San Jose, CA, USA) and CD8b (clone 295/33-25, BD Pharmingen, San Jose, CA, USA). Dead cells were excluded by staining for viability with the fixable viability dye eFluor 450 (eBioscience, Waltham, MA, USA). Intracellular IFNγ was stained with AF647-conjugated antibody (clone P2G10, BD Pharmingen) after fixation and permeabilization of cells using the Cytofix/Cytoperm kit (BD) according to the manufacturer’s recommendations. Cells were analyzed on an LSR Fortessa flow cytometer (BD) and data were analyzed with FlowJo software version X.0.7 (Treestar, Ashland, OR, USA).

### 2.12. Statistical Analyses

Clinical and experimental data were shown as mean or geometric mean and standard deviation as mentioned within the text and in graphical formats. For multiple comparisons between groups, a non-parametrical Kruskal-Wallis test followed by Dunn’s multiple comparisons test was performed. A *p*-value of ≤ 0.05 was considered statistically significant. Area under the curve analysis was performed for each sample analyzed by ELISA. Baseline values were calculated for each analyzed plate using the optical mean density plus three standard deviations of wells incubated without sera. Statistics were performed using Graphpad Prism version 7.00 for Windows, GraphPad Software, La Jolla, CA, USA.

## 3. Results

### 3.1. Vaccination Did Not Result in an Increase of Antibodies against H1 HA Head and Stalk Domains

**Figure 2:**
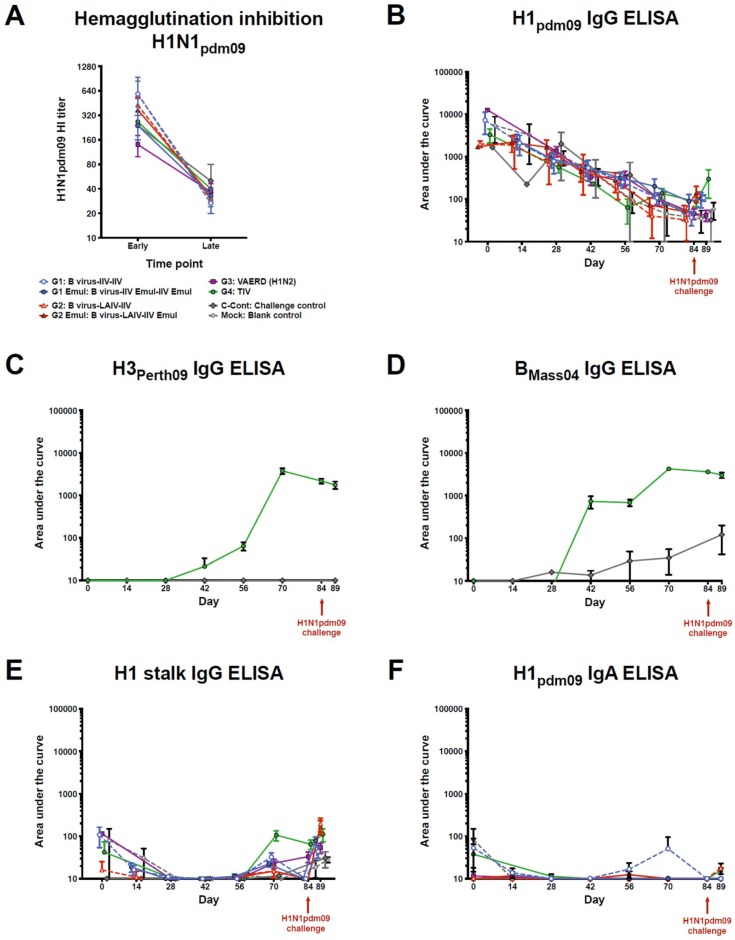
Serology after vaccination and infection. (**A**) Hemagglutination inhibition titers against pH1N1 were measured in the earliest available and the last pre-challenge serum samples from all pigs. The points indicate the mean titers for each group and the error bars show the standard error means. (**B**–**E**) IgG titers were measured by ELISA against different influenza virus HAs over time. Points indicate the mean area under the curve value for each group and the error bars show the standard error means. (**F**) IgA titers were measured by ELISA against pH1 over time. Points indicate the mean area under the curve value for each group and the error bars show the standard error means.

As a first step, we analyzed the serological response of the vaccinated animals. All pigs used in the experiment had high pre-existing maternal antibody titers against pandemic H1N1 on the day of the first vaccination. Independent of the vaccination strategy used, the maternal antibody titers decreased during the course of the vaccination experiment to a hemagglutination inhibition (HI) titer of approximately 1:40 before the viral challenge (Figure 2A). Even animals that received the TIV with the pH1N1 component had a decline in titer despite the expectation that this group should have induced a vaccine-mediated H1 HI response. When anti-H1 IgG titers were measured in an ELISA, similar results were obtained. Initially, animals had very high anti-H1 IgG titers but this reactivity decreased over the time course of the experiment. Interestingly, none of the vaccination strategies (including TIV) resulted in an increase of IgG titers against H1 (Figure 2B). To test if the lack of an anti-H1 antibody increase was due to insufficient immunogenicity of the vaccine or possibly due to pre-existing maternal antibodies against H1, we tested antibody titers against the other components of the TIV—H3 and influenza B virus hemagglutinin (HA) in the TIV vaccination and control groups. The pigs in the TIV group (G4) were all naïve for both components at the beginning of the vaccination experiment, but over time developed high antibody titers against both H3 and influenza B HAs (Figure 2C,D). This indicates that the lack of a vaccine response was specific to the H1-component and probably due to the presence of H1-specific maternal antibodies. A low rise in background IgG levels against influenza B HAs was observed in the challenge control group (Figure 2D).

Candidate universal influenza virus vaccines based on a vaccination strategy with cHAs have previously been shown to potently induce protective titers of broadly cross-reactive antibodies against the conserved HA stalk domain in mice and ferrets [20,21,35,48,49,50]. To test if antibodies against the H1 stalk domain were induced in the vaccinated pigs, their sera were tested by ELISA against a cHA that consisted of an H6 head domain (to which pigs are naïve) and the H1 stalk domain. The experimentally vaccinated pigs showed low HA stalk antibody titers at the start of the experiment, which were most likely part of the maternal antibody repertoire. The titers then decreased to baseline by day 28 and showed a slight increase on day 70 for some groups, but remained very low overall. Interestingly, the titers for all groups increased post H1N1 challenge and this increase was larger compared to the mock controls for some groups, which could indicate a recall response and illustrates a booster effect for this type of antibody by exposure to live virus (Figure 2E).

Since the universal vaccination groups received a live virus prime with an influenza B virus expressing cH9/1 and two groups then received a boost with a cH8/1N1 LAIV, we also measured the induction of serum IgA against H1, which are more commonly associated with mucosal protection [51,52]. The levels of H1-specific IgA were very low on day 0 and reverted to baseline on day 14. This is consistent with a maternal IgG-predominant immunity that was transferred to the pigs. No detectable induction of H1-specific IgA was observed at later time points (Figure 2F).

### 3.2. Virus Replication in the Respiratory Tract

**Figure 3:**
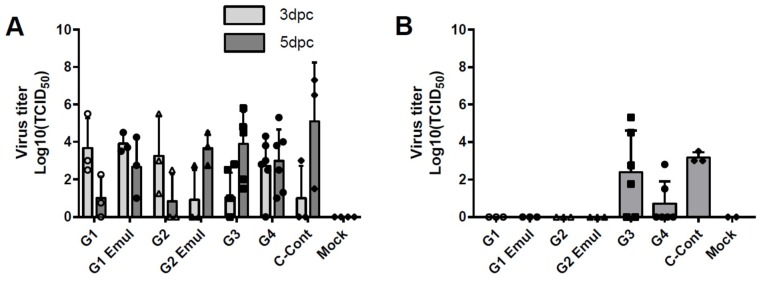
Virus titers of nasal swab and BALF samples. (**A**) Virus titers of nasal swab samples at 3 and 5 dpc. Bars represent means and error bars represent standard deviations (**B**) Virus titers measured in BALF at 5 dpc. Virus titer is presented as TCID_50_/mL. A TCID_50_ was calculated using the Reed and Muench method. Bars represent means and error bars represent standard deviations. Open and filled symbols (△, □, ○ and ◊) were used to show individual data points for the different experimental groups.

To evaluate the protective effect of the universal vaccine approach in terms of virus replication, virus titers in nasal swabs and BALF were measured in the challenged animals. Virus titers in nasal swabs were negative on day 1 post challenge (dpc) in all groups (Table 3). Virus titers were significantly different (*p* < 0.05) between groups in both BALF (5 dpc) and nasal swabs (3 dpc and 5 dpc) as measured with the Kruskal-Wallis test. However, there were no significant differences when comparing the specific groups with each other using the Dunn’s multiple comparisons post test. All the animals, except for the mock challenged animals, had at least one pig with detectable virus titers in nasal swabs at 3 dpc, with variability in titers between animals within the same group (Table 3, Figure 3A). At 5 dpc, virus titers and the number of positive animals per group presented a different pattern between the groups. Groups G1, G1 Emul and G2 showed decreased virus titers of 10, 10^2.67^ and 10^0.83^ TCID_50_/mL, respectively. Moreover, not all animals tested positive for infectious influenza viruses in groups G1 and G2 (Table 2, Figure 3A). The control group C-Cont and groups G2 Emul, G3 (VAERD control) and G4 (TIV) had higher virus titers; 10^5.08^, 10^3.67^,10^3.88^ and 10^2.98^ TCID_50_/mL respectively (Table 2, Figure 3A). No virus was detected in the BALF of any of the experimental universal influenza virus vaccine groups (groups G1, G1 Emul, G2 and G2 Emul). Virus titers of group G3 (VAERD), group G4 (TIV) and the infected control group C-Cont in the BALF averaged 10^2.38^, 10^0.71^ and 10^3.19^ TCID_50_/mL, respectively at 5 dpc (Figure 3B). This suggests complete protection of the lung by the universal vaccine approach despite the absence of significant antibody responses against the stalk domain; however, numbers of animals per group were too small to firmly conclude that this was not caused by experimental variation.

### 3.3. Pathology

**Figure 4:**
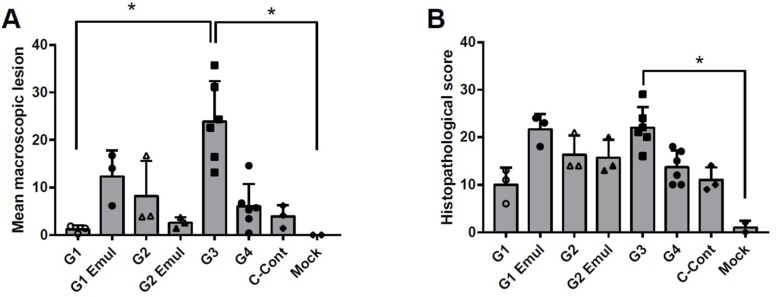
Macroscopic lung lesion scores and histopathology 5 dpc. (**A**) Macroscopic lesions observed in lung tissues. Bars represent means of macroscopic lung lesion scores observed on different tissues of the respiratory tract as described in the text. Error bars represent standard deviations. (**B**) Histopathology scores observed in lung tissues. Bars represent means and error bars represent standard deviations. Open and filled symbols (△, □, ○ and ◊) were used to show individual data points for the different experimental groups. * indicates statistical significant differences between the tested groups (*p* < 0.05) as measured by Dunn’s multiple comparisons test performed after a Kruskal-Wallis test.

The macroscopic lung lesions from the experimental groups except the mock (non-challenged) control group had multifocal coalescing areas with purple-red consolidation that was mild to moderate depending on the group. All experimental groups had on average a relatively low macroscopic lung lesion score when compared to other reports of pigs infected with the pH1N1 virus [44,53]. Lung lesions were present mainly in the cardiac lobe of groups G1 and G2 and in the cardiac lobe and the apical lobe for groups G1 Emul and G2 Emul. The highest percentage of lesions were 10% and 45% for the apical lobe and cardiac lobe, respectively. In contrast, group G3 (H1N2 vaccinated VAERD control group) revealed typical influenza-associated lung lesions in all seven lobes and the cardiac lobe was affected 35–90%, which tracked with prior reports [28]. The mean macroscopic lung scores for each experimental group are shown in Figure 4A. Lung scores for macroscopic lesions were significantly higher for the VAERD group when compared to G1 or the mock control group (Figure 4A, *p* < 0.05 Dunn’s multiple comparisons post test after Kruskal-Wallis test).

**Figure 5:**
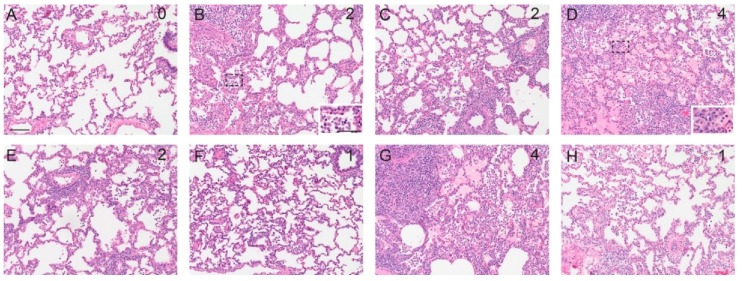
Histological examination of lung pathology with HE stain. Lungs collected at 5dpc were scored for interstitial pneumonia on a scale of 0, no pneumonia, to 4 severe, coalescing to diffuse lymphohistiocytic pneumonia as described in Table 2. The pneumonia score is in the upper right. A representative lung field is shown for (**A**) mock infected control, (**B**) Cont-C, (**C**) G1, (**D**) G1 Emul, (**E**) G2, (**F**) G2 Emul, (**G**) G3 VAERD and (**H**) G4. Bar in A is 100 μm and bar in inset of B is 50 μm.

Lungs collected at 5 dpc were scored for interstitial pneumonia and representative histopathological results from individual groups are presented in Figure 5. The histological score (maximum score = 25) was determined based on parameters described in materials and methods. Group G1 revealed the lowest histological score compared to the other experimental groups and group G1 Emul and group G3 (VAERD) had the highest mean scores (see Table 2, and Figure 4B). Histopathology scores were significantly different between G3 (VAERD) and the mock control group (*p* < 0.05 Dunn’s multiple comparisons post test after Kruskal-Wallis test). All tissues from mock infected, negative control animals were negative for influenza antigen as expected. Lung tissue tested positive from the majority of all animals in all groups with the exception of the infected control group, for which the majority of tracheal samples were positive. IHC tested on various respiratory tissues revealed that the experimental universal influenza vaccine groups exhibited a lower rate of positive cells and tissues when compared to group G3 (VAERD) (Table 4). Specifically, group G1 pigs had no viral antigen in their tracheas and nasal turbinates. Challenge control pigs (C-Cont) had viral antigen in their trachea samples and one out of three nasal turbinate samples.

### 3.4. Cellular Responses Measured after Challenge Reflect Priming by Influenza Virus Vaccination

**Figure 6:**
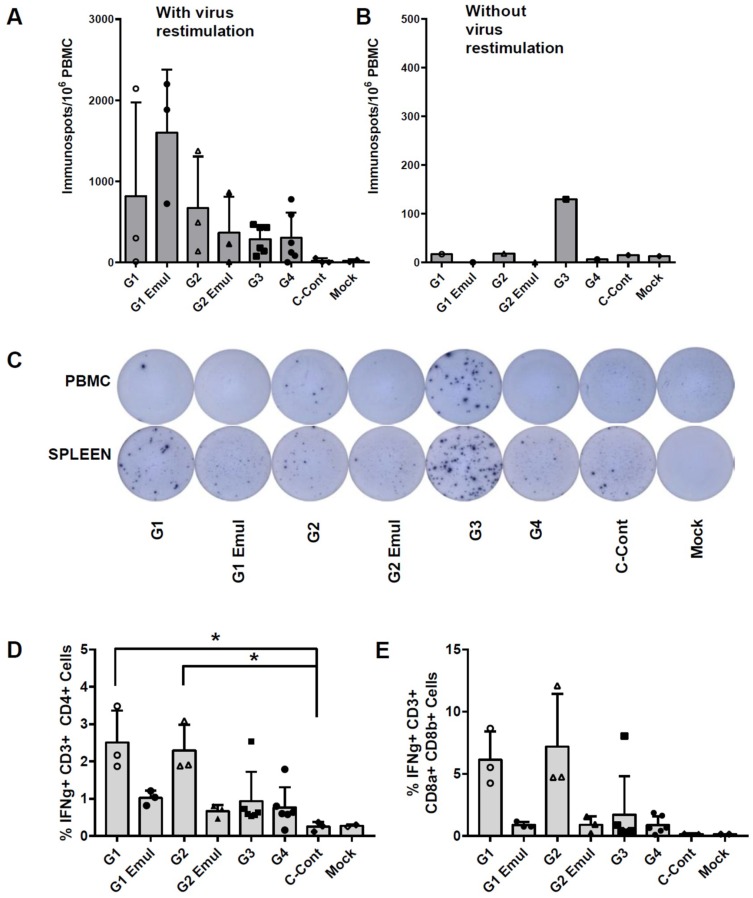
T cell responses at 5 days post challenge. (**A**) IFNγ ELISPOT with PBMCs of individual animals restimulated over night with pH1N1 virus. (**B**) IFNγ ELISPOT with pools of PBMCs that were left over night without restimulation to estimate background levels of IFNγ secretion. (**C**) Images of ELISPOT wells of pooled PBMCs or splenocytes left over night without virus restimulation. (**D**) Percentage of IFNγ+ CD4+ T cells in PBMCs after 8 h of pH1N1 virus restimulation. (**E**) Percentage of IFNγ+ CD8a+ CD8b+ T cells in PBMCs after 8 h of pH1N1 virus restimulation. Bars represent means and error bars represent standard deviations. Open and filled symbols (△, □, ○ and ◊) were used to show individual data points for the different experimental groups. * indicates statistical significant differences between the tested groups (*p* < 0.05) as measured by Dunn’s multiple comparisons test performed after a Kruskal-Wallis test.

Influenza virus-specific T cell responses upon ex vivo virus restimulation were measured in peripheral blood mononuclear cells (PBMCs) and splenocytes 5 dpc by interferon γ (IFNγ) enzyme-linked immunospot (ELISpot) assay and flow cytometry. Pools of non-restimulated PBMCs and spleen cells were used as technical controls to estimate background levels. T cell responses around background levels were also measured in the blood and spleen of animals that received the challenge virus without prior vaccination or non-challenged animals. From all tested vaccine groups, adjuvanted universal vaccine groups in which the inactivated vaccine was administered twice (G1 Emul) resulted in the highest numbers of IFNγ immunospots after virus restimulation (Figure 6A). The VAERD control group G3 and group G4 (TIV vaccine) showed similar numbers of immunospots, which was on average lower than in animals that received the experimental universal influenza virus vaccine (groups G1, G1 Emul, G2, G2 Emul). Pools of PBMCs and splenocytes from animals vaccinated with the H1N2 vaccine had more IFNγ-producing cells compared to the other experimental groups as detected by ELISpot in the absence of virus restimulation (Figure 6B,C), which suggests a higher baseline activation of circulating IFNγ-producing cells in pigs that experience VAERD. After infection, T cell populations in PBMCs were measured by flow cytometry after intracellular cytokine staining of virus stimulated cells. Groups G1 and G2 showed the highest percentage of IFNγ+ CD4+ and IFNγ+ CD8a+ CD8b+ T cells (Figure 6D,E). Groups G1 Emul, G2 Emul, G3 and G4 had similar percentages for IFNγ+ T cells, and these were always higher than the unvaccinated control groups (groups C-Cont and mock).

## 4. Discussion

Current influenza virus vaccines consist of two influenza A (H1N1 and H3N2 subtype) and one or two influenza B virus strains. The inclusion of multiple influenza virus strains into the vaccine is needed since they can co-circulate in the human population thereby causing severe morbidity and mortality, and challenging public health systems worldwide. Besides the requirement to include multiple virus strains, antigenic drift and shift necessitates reformulation of currently licensed influenza virus vaccines on an almost yearly basis in order to antigenically match the vaccines as best as possible with the circulating virus strains. The strategy of a universal influenza virus vaccine that we used for this study focuses on the conserved stalk domain of HA as immunogen for broad cross-protection [7,54,55,56]. To date, the concept of an HA-based universal influenza virus vaccine has been tested with different approaches, most of them rely on the binding and neutralizing activity of stalk-reactive antibodies [15,20,37,57,58]; this is inspired by protection against influenza virus infection seen in different preclinical models provided by monoclonal antibodies against the stalk domain of HA. Since the report of the first monoclonal antibody against the stalk domain, C179, with broad binding and neutralization against group 1 HA expressing viruses that was published in 1993 [59], many monoclonal antibodies with protective effect against influenza virus have been described and are a potential safe and efficacious tool in the battle against influenza [16,19,60,61,62,63,64]. HA-based universal influenza virus vaccine approaches resulted in the induction of broadly cross-reactive antibodies and protection in mouse, ferret and non-human primate models [20,21,35,49,57,58].

In this study, we assessed the efficacy of a cHA-based universal influenza virus vaccine regimen in a commercial pig model in the presence of maternal antibodies against pH1N1 virus. In humans, maternal antibodies are believed to contribute to the protection of the infant during the first 6 months of life, before the first influenza vaccination is given [65,66]. Maternal antibodies will decrease over time and it is currently unclear how they affect vaccine responses. In this experiment, we observed that maternal antibodies against the challenge strain decreased over time to an HI titer of approximately 40. This did not protect animals from experimental pH1N1 infection. We tested different vaccination strategies and formulations; the experimental universal vaccine was given as a combination of cHA expressing LAIV followed by cHA-based IIV or IIV followed by IIV and either with or without adjuvant (see Table 1). None of the vaccine approaches, including the classical, matched TIV vaccine (group G4), resulted in an increase of H1-specific IgG or IgA responses. Since animals that received the TIV vaccine (group G4) did mount an antibody response against the H3 and influenza B component of the vaccine, it is possible that the presence of H1-specific maternal antibodies at early vaccination time points resulted in poor induction of de novo H1-specific antibody responses against both HA head and stalk domains. This is in contrast to other animal models, where the cHA-based vaccine strategy did effectively induce anti-stalk antibodies, in the absence of maternal antibodies against HA. An experimental group that lacks maternal antibodies was not available at the start of the experiment; this would allow to address the role of such antibodies on the induction of HA stalk antibodies in the pig model. Maternal HA stalk-specific antibody levels were close to the limit of detection of the assay and no difference between vaccine and control groups were observed around the day of challenge with pH1N1.

Non-adjuvanted experimental universal influenza virus vaccine groups (G1 and G2) were protected from pH1N1 virus challenge by reducing virus replication in nasal swabs by 5 dpc and sterilizing immunity in BALF, despite the lack of a robust anti-stalk response. Adjuvanted universal vaccine groups (G1 Emul and G2 Emul) revealed a complete elimination of virus titers in BALF. However, virus titers in nasal swabs were comparable to the TIV group G4 but lower than in the unvaccinated control group C-Cont by 5 dpc.

As expected, virus titers in H1N2 vaccinated pigs (VAERD control group, G3) were only slightly lower than those of non-vaccinated infected animals (C-Cont) in both nasal swabs and BALF. Compared to non-vaccinated animals (C-Cont), VAERD animals had higher scores for pathology based on macroscopic and microscopic lesions. Therefore, we conclude that we reproduced the enhanced pneumonia which is typical for VAERD [28] by vaccinating with an Emulsigen adjuvanted swine H1N2 vaccine followed by infection with pH1N1 virus in the presence of maternal antibodies to pH1N1. Importantly, this group had similarly low levels of anti-stalk antibodies as compared to all other vaccinated groups. This finding is in contrast to the suggestion that antibodies which target a linear epitope in the stalk of HA, would be responsible for VAERD in a similar swine-influenza challenge model [34], since VAERD did not seem to correlate with the levels of HA stalk-specific antibodies in the present study.

One experimental universal vaccine approach given as adjuvanted IIV prime-boost regimen (group G1 Emul) results in similar histological scores as the VAERD control group G3 (Figure 4B, Figure 5 and Table 3), despite complete virus control in the lung (Figure 3B) and despite lower scores for macroscopic lesions than group G3. This suggests a negative effect of the specific oil-in-water adjuvant used in this study. Based on the histological score, such an adjuvant effect was not seen for group G2 Emul, in which animals received an LAIV followed by adjuvanted IIV, which is in line with a superior heterologous protection by LAIV seen in earlier studies [67,68].

Induction of influenza virus-specific T cell responses can correlate with protection in the swine influenza model in the absence of neutralizing antibody [69]. However, T cell activation has also been reported to be associated with VAERD in pigs [27]. This illustrates that both quality and quantity of T cell responses are important factors to consider. Both LAIV and the use of an adjuvant can promote the induction of T cell responses. Interestingly, group G1 Emul has high numbers of IFNγ immunospots as measured by ELISpot using virus restimulation of PBMCs collected at 5 dpc. However, this is not seen for the VAERD control group G3. The VAERD group had more IFNγ immunospots in pools of PBMCs or splenocytes compared to the other experimental groups when cells are not restimulated with the virus. One explanation might be that in animals experiencing VAERD, increased damage of lung tissue results in viremia, which leads to restimulation of T cells in the blood and also in ex vivo restimulation cultures of PBMCs and splenocytes. Another possibility is that the source of IFNγ in these ex vivo cultures is not derived from T cells, but from natural killer (NK) cells, natural killer T (NKT) cells or a myeloid cell type that is already activated and does not require restimulation with the virus in order to produce IFNγ. A more extensive cytokine analysis at different time points post infection would be important to determine the involvement of different cytokines in VAERD. When we focused on the phenotype of IFNγ-producing cells by flow cytometry, the unadjuvanted universal vaccine approaches (groups G1 and G2) had the highest percentages of IFNγ+ CD4+ and CD8a+ CD8b+ T cells which in turn correlated with optimal virus control by 5 dpc. Both adjuvanted universal vaccine groups (G1 Emul and G2 Emul) had similar levels of IFNγ-producing T cells as G3 (VAERD control) and G4 (TIV control). De novo induction of T cell responses by the challenge virus most likely requires more than 5 days in order to be detectable in PBMC by ELISpot or flow cytometry. Therefore, T cell responses above background levels measured at 5 dpc are likely the result of vaccination, eventually boosted by the virus challenge. All vaccine groups have higher T cell levels than naive challenged animals (C-Cont). This suggests that vaccination indeed resulted in efficient T cell priming.

The N1 neuraminidase is shared by the vaccine strains and challenge virus. Therefore, we cannot exclude that N1-specific immune responses induced by vaccination contributed to protection after virus challenge. The contribution of neuraminidase- and HA head-specific antibodies to protection is the focus of future research.

In summary, we showed that a universal influenza virus vaccine based on the conserved stalk of HA can provide low level protection against challenge with the pH1N1 virus in pigs with maternal immunity despite the absence of a robust anti-stalk response in this model. Best protection in terms of virus reduction was obtained by sequential vaccination with live influenza B virus expressing chimeric H9/1 HA followed by two unadjuvanted inactivated virus vaccines containing cH8/1 and cH5/1 antigens, respectively, although there was too much experimental variability among pigs within groups to come to a firm conclusion. In the absence of a robust anti-stalk response, the induced protection is likely caused by a T cell response with potential contribution from low levels of antibodies. We also showed that the induction of VAERD by vaccination with an adjuvanted inactivated H1N2 vaccine followed by challenge with pH1N1 was not correlated with stalk-reactive antibodies that target the HA2 subdomain of HA. The protection and absence of VAERD in pigs that received the universal virus vaccine are in line with what we reported before for a similar study we performed in the ferret model, where the universal influenza vaccine did induce high stalk HA antibody titers [48].

## 5. Conclusions

A universal influenza virus vaccine approach in the presence of maternal antibodies resulted in a partial protective effect when animals were challenged with pH1N1 virus, despite the lack of induction of HA stalk-specific antibodies. In contrast, immunization with an adjuvanted H1N2-based vaccine followed by pH1N1 challenge led to enhanced respiratory disease without statistical significantly higher stalk-specific antibodies compared to the other groups. These observations suggest that HA stalk-specific antibodies are not the cause of vaccine-associated enhanced respiratory disease in this pig challenge model.

## Figures and Tables

**Table 1:** Experimental groups.

Group	Vaccine Composition			No. of Pigs
	Prime	Vaccine #1	Vaccine #2	
G1	B-cH9/1 ^a^	IIV cH8/1 ^b^	IIV cH5/1 ^d^	3
G1 Emul	B-cH9/1	IIV cH8/1 with adjuvant	IIV cH5/1 with adjuvant	3
G2	B-cH9/1	LAIV cH8/1 ^c^	IIV cH5/1	3
G2 Emul	B-cH9/1	LAIV cH8/1	IIV cH5/1 with adjuvant	3
G3	-	H1N2 inactivated whole virus with adjuvant		6
G4	-	TIV containing H1N1 with adjuvant		6
C-Cont	Challenged control			3
Mock	Non-vaccine & non-challenge (Mock)			2

^a^ Chimeric influenza B virus with H9 globular head domain on top of H1 stalk domain; ^b^ 15 μg of inactivated chimeric influenza virus A with H8 globular head domain on top of H1 stalk domain; ^c^ 32 HAU of live attenuated chimeric influenza virus A with H8 globular head domain on top of H1 stalk domain; ^d^ 15 μg of inactivated chimeric influenza virus A with H5 globular head domain on top of H1 stalk domain.

**Table 2:** Histopathology score descriptions.

Parameter	HP Score				
	0	1	2	3	4
Lung % involvement: Epithelial changes and inflammation	None	1–25%	26–50%	51–75%	Greater than 75%
Peribronchiolar cuffing by lymphocytes	None	Mild, loosely formed cuffs of lymphocytes	Moderate, well-formed cuffs of lymphocytes	Prominent thick well-formed cuffs of lymphocytes	
Degree of interstitial pneumonia (IP)	None	Mild, focal to multifocal IP	Moderate, locally extensive to multifocal IP	Moderate, multifocal to coalescing IP	Severe, coalescing to diffuse IP
Tracheal epithelial changes & Nasal cavity epithelial changes	None	Early epithelial changes with focal to multifocal loss of cilia and epithelial degenerative changes	Mild epithelial flattening with loss of cilia and goblet cells	Moderate epithelial flattening with decreased thickness of respiratory epithelium, loss of cilia and goblet cells	Flattened epithelium with areas of mucosa covered by a single layer of cuboidal epithelium and epithelial loss (necrosis)
Degree of rhinitis	None	Mild	Moderate	Severe	
Degree of tracheitis	None	Mild	Moderate	Severe	

HP: histophatologic. The highest possible score is 25.

**Table 3:** Number of virus positive animals per group.

	DPC	G1	G1 Emul	G2	G2 Emul	G3	G4	C-Cont	Mock
Vaccine Composition		B virus	B virus	B virus	B virus				
		IIV	IIV Emul	LAIV	LAIV	VAERD	TIV		
		IIV	IIV Emul	IIV	IIV Emul				
Nasal swab	0	0/3	0/3	0/3	0/3	0/6	0/6	0/3	0/2
	1	0/3	0/3	0/3	0/3	0/6	0/6	0/3	0/2
	3	3/3	3/3	3/3	1/3	3/6	5/6	1/3	0/2
	5	2/3	3/3	1/3	3/3	6/6	6/6	3/3	0/2
BALF	5	0/3	0/3	0/3	0/3	4/6	2/6	3/3	0/2

DPC = Day Post Challenge.

**Table 4:** Immunohistochemistry and histological score results.

Experimental Groups	Immunohistochemistry (IHC): Positive/Total Samples							
	G1	G1 Emul	G2	G2 Emul	G3	G4	C-Cont	Mock
Lung	2/3	3/3	2/3	2/3	6/6	6/6	1/3	0/2
Trachea	0/3	2/3	2/3	3/3	6/6	5/6	3/3	0/2
Nasal turbinates	0/3	2/3	0/3	1/3	3/6	4/6	1/3	0/2

## Abbreviations

BALF	bronchio-alveolar lavage fluid
cHA	chimeric hemagglutinin
CPE	cytophathic effect
DPC	days post challenge
Emul	Emulsigen adjuvant
H&E staining	hematoxylin and eosin staining
HA	hemagglutinin
HI	hemagglutination inhibition
ICS	intracellular cytokine staining
IFN	interferon
Ig	immunoglobulin
IHC	immunohistochemistry
IIV	inactivated whole virus influenza vaccine
LAIV	live attenuated influenza virus vaccine
MDCK	Madin Darby canine kidney
MEM	minimal essential medium
NP	nucleoprotein
PBMCs	peripheral blood mononuclear cells
PFU	Plaque forming units
TCID50	tissue culture infectious dose 50
TIV	trivalent inactivated virus vaccine
TPCK	tosyl phenylalanyl chloromethyl ketone
VAERD	vaccine-associated enhanced respiratory disease
