# Erratum: Sunwoo, S.-Y. et al. A Universal Influenza Virus Vaccine Candidate Tested in a Pig Vaccination-Infection Model in the Presence of Maternal Antibodies. *Vaccines* 2018, *6*(3), 64

**DOI:** 10.3390/vaccines6040080

**Published:** 2018-11-27

**Authors:** Sun-Young Sunwoo, Michael Schotsaert, Igor Morozov, Anne Sally Davis, Yuhao Li, Jinhwa Lee, Chester McDowell, Philip Meade, Raffael Nachbagauer, Adolfo García-Sastre, Wenjun Ma, Florian Krammer, Juergen A. Richt

**Affiliations:** 1 Department of Diagnostic Medicine & Pathobiology, College of Veterinary Medicine, Kansas State University, Manhattan, KS 66506, USA; sunwoosy@gmail.com (S.-Y.S.); imorozov@vet.k-state.edu (I.M.); asally@vet.k-state.edu (A.S.D.); yuhaoli@wustl.edu (Y.L.); jinhwa@vet.k-state.edu (J.L.); cdmcdow@vet.k-state.edu (C.M.); 2 Department of Microbiology, Icahn School of Medicine at Mount Sinai, New York, NY 10029, USA; michael.schotsaert@mssm.edu (M.S.); philip.meade@icahn.mssm.edu (P.M.); raffael.nachbagauer@mssm.edu (R.N.); Adolfo.Garcia-Sastre@mssm.edu (A.G.-S.); 3 Graduate School of Biomedical Sciences, Icahn School of Medicine at Mount Sinai, New York, NY 10029, USA; 4 Global Health and Emerging Pathogens Institute, Icahn School of Medicine at Mount Sinai, New York, NY 10029, USA; 5 Department of Medicine, Icahn School of Medicine at Mount Sinai, New York, NY 10029, USA

In the published article, “A Universal Influenza Virus Vaccine Candidate Tested in a Pig Vaccination-Infection Model in the Presence of Maternal Antibodies. *Vaccines* **2018**, *6*(3), 64. doi:10.3390/vaccines6030064 [1]”, the contract number in the funding section was incorrect.

In the funding section, the contract number HHSN266200700006C should be replaced with HHSN272201400006C.

The authors would like to apologize for any inconvenience caused to the readers by this change. The manuscript will be updated and the original copy will remain online on the article webpage.
